# Generation and Processing of Simulated Underwater Images for Infrastructure Visual Inspection with UUVs

**DOI:** 10.3390/s19245497

**Published:** 2019-12-12

**Authors:** Olaya Álvarez-Tuñón, Alberto Jardón, Carlos Balaguer

**Affiliations:** Department of Systems and Automation Engineering, Roboticslab, University Carlos III Madrid, Av. Universidad 30, E-28911 Leganés, Madrid, Spain; ajardon@ing.uc3m.es (A.J.); balaguer@ing.uc3m.es (C.B.)

**Keywords:** Underwater robotics, underwater image simulation, style transfer, underwater image formation modelling

## Abstract

The development of computer vision algorithms for navigation or object detection is one of the key issues of underwater robotics. However, extracting features from underwater images is challenging due to the presence of lighting defects, which need to be counteracted. This requires good environmental knowledge, either as a dataset or as a physic model. The lack of available data, and the high variability of the conditions, makes difficult the development of robust enhancement algorithms. A framework for the development of underwater computer vision algorithms is presented, consisting of a method for underwater imaging simulation, and an image enhancement algorithm, both integrated in the open-source robotics simulator UUV Simulator. The imaging simulation is based on a novel combination of the scattering model and style transfer techniques. The use of style transfer allows a realistic simulation of different environments without any prior knowledge of them. Moreover, an enhancement algorithm that successfully performs a correction of the imaging defects in any given scenario for either the real or synthetic images has been developed. The proposed approach showcases then a novel framework for the development of underwater computer vision algorithms for SLAM, navigation, or object detection in UUVs.

## 1. Introduction

The development of computer vision algorithms for underwater robotic platforms is gaining attention thanks to the great advances that vision capabilities have recently experienced. They provide a wide range of utilities, from marine investigation [1,2], to archaeology [3] or structures monitoring [4,5]. Particularly interesting is the use of computer vision for underwater robot localization [6], for which expensive sensors based on acoustic technologies have been mainly used to date [7].

**Figure 1:**
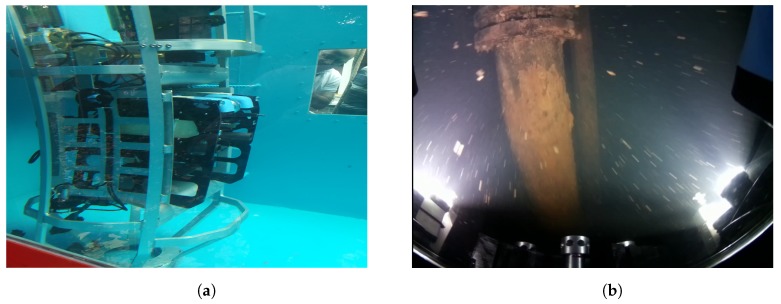
Deployment of the STAMS project underwater robot. (**a**) The modified version of the BlueROV2. (**b**) Capture obtained by the BlueROV2 camera in a flooded mine shaft during one of the STAMS field trials.

The present work is framed within the STAMS (Long-Term Stability Assessment and Monitoring of Flooded Shafts) European project, under the Research Fund for Coal and Steel (RFCS). The main objective of the STAMS project is to implement solutions to monitor the lining stability of flooded shafts for long periods of time. A modified version of the underwater robot BlueROV2 has been used as the robotic platform for this project (see Figure 1a), equipped with low-cost sensors such as a camera and an inertial sensor. This leads to the deployment of the robot being based on computer vision approaches for inspection and navigation.

However, underwater images suffer from poor contrast and blurring due to the exponential attenuation and scattering caused by light transmission conditions. Moreover, as seen in Figure 1b, the use of artificial lights provides a non-uniform illumination of the scene, and bright spots for each source of light surrounded of a poorly illuminated area. This negatively affects to the development of computer vision algorithms, making necessary to perform a prior processing of the images.

The main limitation to the development of underwater computer vision algorithms is the lack of large enough databases, especially for deep learning approaches, where usually synthetic datasets are generated [8,9]. There are some datasets for object detection [10,11], restoration [12] or visual navigation [13]. However, the imaging conditions strongly vary between different environments, since the attenuation and scattering suffered by light in water depend on factors such as water temperature, salinity or suspended particles [14].

A similar problem occurs in simulation. The available open-source underwater simulators are UWSim (UnderWater Simulator) [15] and UUV (Unmanned Underwater Vehicle) Simulator [16]. UWSim allows for visualization of a virtual underwater scenario, as well as simulated sensors and a control interface for underwater vehicles. Similarly, the UUV Simulator models underwater hydrostatic and hydrodynamic effects and sensors for underwater robots. However, both simulators are mainly meant for robot dynamics, and thus very limited in terms of imaging.

All these underwater conditions pose a research problem that highlights the need for data and tools to ease the development of computer vision algorithms under these constraints. Moreover, their integration within a robotics simulation will allow a faster and easier development of these algorithms for a wide range of robotic applications.

This work presents various contributions for underwater images generation and processing. The main contribution lies in the development of an open-source tool for underwater imaging simulation, particularly in the use of style transfer. The main interest and novelty on the use of style transfer comes from the possibility of using a single training image to obtain a reliable simulation of any desired underwater environment, since this technique has been mainly applied to design and entertainment applications. Moreover, an image enhancement algorithm has been developed which optimizes the extraction of features from the image for any given environment, which is a key issue in localization, navigation or tracking algorithms.

**Figure 2:**
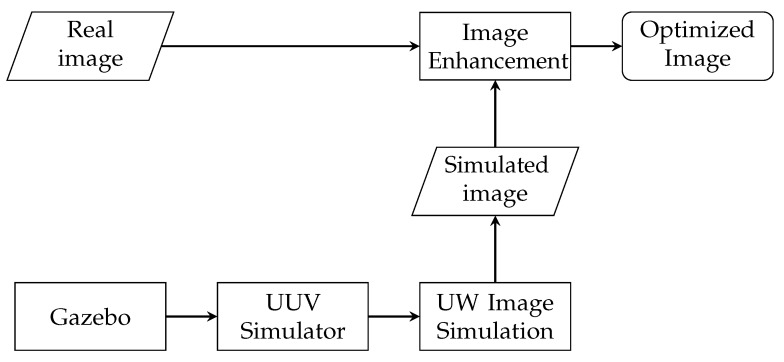
The proposed framework for simulation and development of underwater computer vision algorithms.

This whole ensemble represents then a novel framework for the development of underwater computer vision algorithms, and it is additionally integrated within the UUV Simulator for their development in underwater robots. The proposed framework is summarized in Figure 2: the image provided by the UUV Simulator is processed to emulate the underwater imaging conditions, and the enhancement algorithm can be applied either to a simulated or a real image.

The present work is structured as follows: First, the related works on underwater image formation, style transfer and image enhancement are introduced in Section 2. Then, the proposed method for underwater image simulation and the image enhancement algorithm developed within the framework are explained in Section 3 and Section 4. The results and conclusions are shown in Section 5 and Section 6.

## 2. Related Works

Test and experimentation with UUVs is a challenging task due to the inaccessibility of their work environment. For that reason, there has been work on developing simulators for underwater vehicles, as surveyed in [17,18].

UWSim [15] and SubSim [19] are the only known recent simulators exclusively developed for underwater vehicle simulation. Both provide a simulation of the rigid body dynamics and of underwater sensors such as sonar or pressure sensors. Moreover, UWSim simulates suspended particles and different water colors, as well as water surface effects such as waves and reflections. However, there has not been recent development on any of both.

The current trend is the development of general-purpose robotic simulators such as Gazebo [20] and V-REP [21], that allow the integration of plugins for specific tasks. They can simulate a wide variety of sensors, and support different physics engines. The UUV Simulator [16] is a plugin for Gazebo that allows underwater simulation. It extends the physics engine to simulate the rigid body dynamics underwater, as well as some typical underwater sensor models. This simulator is however more focused on modelling the physical fidelity rather than the imaging conditions. There have been previous works on using V-REP as an underwater simulator too [22], but again the development of plugins is focused on simulating the hydrodynamic effects.

However, none of these simulators has integrated an underwater imaging modelling realistic enough to develop underwater computer vision algorithms. Hereafter, some related works on modelling the underwater image formation are presented, as well as the previous works on style transfer that are related to the current research problem.

### 2.1. Underwater Image Formation Modelling

The numerical modelling of the underwater image formation presents various challenges, such as accounting the numerous lighting components that exist caused by camera geometry and the optical properties of water. The main model which has been widely used for image formation modelling is based on the linearized formula proposed by Jaffe [23]. Further work has been made in modelling the underwater image formation for computer simulation. However, the main application field of such models is underwater image dehazing [24,25,26], where the model of the image formation is estimated to reverse the defects in the image. The work in [27], revises the Jaffe’s underwater image formation model [23] by adding more factors to the scattering coefficients. A modelling for computer simulation is performed in [28] which extends the model presented by Jaffe. It considers the distortion effects caused by the camera housing thickness, besides the mentioned light propagation.

All previous works model the image formation according to the attenuation and scattering coefficients defined by the light transmission equations.

**Figure 3:**
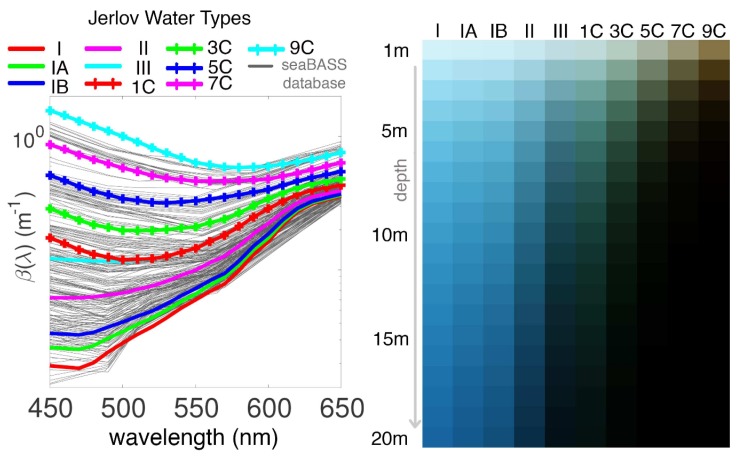
Left: Jerlov water types based on the attenuation coefficients β(λ). Types I-III are oceanic waters, while those suffixed with ‘C’ represent coastal waters with increasing turbidity from 1 to 9. Right: appearance of a white surface viewed from various depths in the different water types. Image courtesy of Akkaynak, D. et al. [14].

There has been work done on modelling those coefficients [14], which strongly vary with the water types. These types, in turn, depend on the lighting and environmental conditions, the concentration of organic and inorganic substances, bathymetry among other factors. A classification of the different water types is shown in Figure 3. The estimation of these parameters requires performing oceanography studies which normally consist of gathering data at different seasons, depths, and water types [29], and with different cameras [30]. To avoid the need for performing such studies, a novel approach based on style transfer is proposed.

### 2.2. Style Transfer

The idea behind Style Transfer is to change the style of a certain input, this is the *how*, without changing its content, this is the *what* it is. The first works to introduce these terms can be found in the area of language processing, concretely in the area of character recognition, separating style and content with bilinear models [31]. Here, the content is defined as the letters, while the style is the actual font or calligraphy.

With the emerge of Deep Neural Networks (DNN), pretrained Deep Neural architectures with high levels of performance in different classification problems, became available for the science community. The VGG (Visual Geometry Group) network [32] was used by Gatys et al. [33] to obtain stylized images using this network inside an iterative optimization process. Here, the content of an image is defined as the outputs of the high-level layers of the VGG network using that image as the input. The style is defined using a Gram Matrix. Using as input a content and a style image, the optimization process generates a third image that minimizes the content and style error with respect to the two input images.

The algorithms used in [34,35] provide comparable results to those proposed by Gatys et al. [33] at a higher speed. The fast algorithm is based on perceptual loss functions dependent on high-level features from a pretrained loss network.

Style transfer has been mainly used for social communication and entertainment, as a tool for people creating their own artworks, for video stylizing and design creation [36]. However, there are no previous approaches that rely on the use of style transfer for underwater image simulation. Deep learning techniques in the context of underwater robots have been mainly used with an opposite goal in mind: underwater color correction. In [37] a CNN (Convolutional Neural Network) is trained to estimate the ambient light and thus to dehaze the image. A more similar approach to style transfer is faced in [38] where the cross-domain relations between air and underwater images are learnt. The semantic color is learnt while preserving the content and structure of the image. The main interest of these algorithms relies on the use a weakly supervised model. A similar problem of generating synthetic underwater images is faced in [9], by using GANs (Generative Adversarial Networks) for modelling the backscatter, the attenuation, and the camera model. However, this solution simulates a specific survey site.

### 2.3. Underwater Image Enhancement

Underwater image processing has been addressed from two points of view: image restoration or image enhancement. Image restoration aims to recover an image with the degradation model of the image formation. This requires the knowledge of the physical model that describes the light propagation, and a depth estimation of the scene objects [23,39]. There are methods based on the blurriness prior, such as IBLA (Image Blurring an Light Absorption), developed by Peng and Cosman [40] which corrects the blurriness according to the estimated background light and scene depth. ULAP (Underwater Light Attenuation Prior) [41] estimates the depth of the scene elements according to the attenuation prior of each color channel. In the work of Li et al. [42], a single underwater image restoration (referred in this paper as SUIR) has been performed. It is based on the Gray-World theory, which assumes that the average value of object color in an ideal image is gray.

On the other hand, image enhancement techniques do not rely on any physical model, and thus do not require any prior knowledge of the environment. They can be based on the frequency components of the image [43], color contrast equalization [44], or color balance [30]. RGHS (Relative Global Histogram Stretching) [45] and RD (Rayleigh Distribution) [46] perform both contrast and color correction. The recent advances in deep learning have also been applied for both restoration and enhancement techniques [37,38].

## 3. Underwater Image Simulation

### 3.1. Background

This work aims to develop a platform for the testing of underwater computer vision algorithms in robotic platforms.

Previous methods commonly define the image intensity $I_{c}$ at each pixel coordinate x=(u,v) in each color channel c∈{R,G,B} as the sum of three components: and attenuated signal and two scattering components:

(1) $$I_{c}\left(x\right)=D_{c}\left(x\right)+F_{c}\left(x\right)+B_{c}\left(x\right)$$

The direct component *D* is the attenuated signal from the object (without scattering), the forward scattering *F* is the light from the object which reaches the camera with small angle scattering, and the backscattering component *B* is the degradation in color and contrast caused by the water scattering effect, where the light does not come from the object. The direct component is exponentially attenuated as:

(2) $$D_{c}\left(x\right)=I_{object}\left(x\right)e^{-a_{c}Z}$$

where $I_{object}\left(x\right)$ is the radiance of the object at point *x* with no underwater effects, $a_{c}$ is the attenuation coefficient and *Z* the distance between the point *x* and the camera.

The blurry effect of the forward scatter component can be modelled by convolution with a point spread function (PSF), as seen in [24,25,26]:

(3) $$\begin{array}{cc} F_{c}\left(x\right) & =D_{c}\left(x\right)*S\left(x\right) \end{array}$$

(4) $$\begin{array}{ccc} \mathrm{with}\;S(x)\;\mathrm{the}\;\mathrm{PSF} & S(x) & =\left[e^{-GZ}-e^{-a_{c}Z}\right]F^{-1}\left(e^{-BZf}\right) \end{array}$$

*G* and *B* are empirical attenuation values, $F^{-1}$ represents the Inverse Fourier Transform and *f* is the frequency variable of *x*. The backscattered light is scattered by suspended particles

(5) $$B_{c}\left(x\right)=B_{\infty }\left(1-e^{-a_{c}Z}\right)$$

with $B_{\infty }$ the radiance of the background light.

Combining Equations (2), (4) and (5) into Equation (1), the image formation model can be finally expressed as:

(6) $$I_{c}\left(x\right)=\left[I_{object}\left(x\right)e^{-a_{c}Z}\right]*S\left(x\right)+B_{\infty }\left(x\right)\left(1-e^{-a_{c}Z}\right).$$

It can be seen that these methods involve many different coefficients for the transmission components of the underwater image formation model. This requires a good knowledge of the environment to simulate, a situation that the present work aims to overcome.

### 3.2. Proposed Method for Underwater Image Simulation

The proposed solution works with the image provided by the UUV Simulator [16]. It has been selected as it is open source and allows the integration between the image simulation and the underwater robot simulation. The UUV’s camera module post-processes the image according to the following exponential attenuation model:

(7) $$I_{c}\left(x\right)=I_{object}\left(x\right)e^{-a_{c}Z}+B_{\infty }\left(1-e^{-a_{c}Z}\right)\;\;\forall \;c\in \left\{R,G,B\right\}$$

where $I_{object}\left(x\right)$ is the original intensity at color channel *c*, $I_{c}\left(x\right)$ the attenuated intensity value, $a_{c}$ the attenuation parameter of light, $b_{c}$ the background light on channel *c* and *Z* the distance from the object to the pixel in the direction of the ray.

However, this is not enough to provide a reliable simulation of the imaging conditions underwater. The proposed solution for underwater image simulation combines the UUV Simulator image processing with the linearized formula proposed by Jaffe [23] and Style transfer techniques, to obtain a more realistic scenario. The image processing consists of three steps:

1. Forward scattering according to Jaffe’s formula.
2. Style transfer of the desired water conditions with the trained models.
3. Haze addition, as caused by a source of artificial light.

This image processing algorithm has been implemented as a ROS (Robot Operating System) node in which all the parameters for the different steps can be configured, and it is publicly available in Github (https://github.com/olayasturias/uw_img_sim).

In this section, each processing step of the algorithm (as shown in Algorithm 1) is explained in detail.

#### 3.2.1. Forward Scattering

The forward scattering causes a blurry effect which increases with distance, due to the small angle of the light reflected from the object to the camera. This scattering effect is modelled as a convolution with a point spread function (PSF), which varies off-axis as the attenuation and spreading increase with distance. The PSF describes the spreading of a very thin beam in water, redistributing the light in the image plane according to the proposed function S(x):

(8) $$\begin{array}{cc} F_{c}\left(x\right) & =I_{c}\left(x\right)*S\left(x\right) \end{array}$$

(9) $$\begin{array}{ccc} \mathrm{with} & S(x) & =e^{-BZ} \end{array}$$

**Figure 4:**
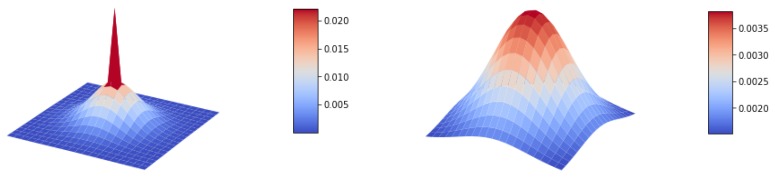
Kernel shape for forward scattering simulation according to the proposed point spread function for a 3 m distance image point (left) and a 50 m distance image point (right).

**Figure 5:**
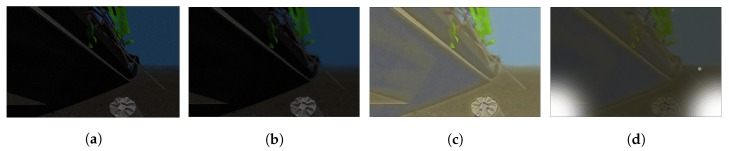
Example of each processing step on an input image. (**a**) Original image from the UUV Simulator. (**b**) Forward-scattered image. (**c**) Result of applying style transfer to the forward-scattered image. (**d**) Addition of two light sources on each of the bottom corners.

The radiance from the object $I_{object}$ at point *x* degrades the details in the image by blurring it according to the constant *B* and the distance *Z*. The plot with the shape of the resulting kernel obtained by applying the previous function at different distances can be seen in Figure 4, where it can be appreciated how the spreading increases with distance. An example of an image blurred with the given PSF is shown in Figure 5b.

#### 3.2.2. Style Transfer for Water Modelling

As previously stated in Section 2.1, the attenuation coefficients for each of the light wavelengths vary depending on the water conditions, affecting the contrast and appearance of the image. A model of each of the water conditions would need then to be computed for the simulation, requiring performance of in situ measurements for each case. Here, the use of style transfer techniques is proposed as an alternative to this modelling step, as it only requires a single image of the underwater conditions to obtain a model for the simulation.

Style transfer from one image onto another is based on deep-convolutional networks pretrained for image classification. Convolutional Neural Networks are the most powerful class of Deep Neural Networks for image processing. Each layer can be seen as a conjunction of image filters that extract a certain feature and thus form the so-called feature maps. When these networks are trained on object recognition, its higher layers capture the high-level content, with their feature responses being referred as the content representation.

The style representation is obtained by extracting the texture information, without considering the global arrangement. Reconstructions from style features produce textured versions of the input image, with size and complexity increasing along the hierarchy.

Style transfer is based on the ability to separate content and style representations. This allows the mixing of two different images and produce a new image by matching the content representation of one image with the style representation of the other. How well this combination is done depends on the loss function. Emphasis can be done on either content or style.

**Figure 6:**
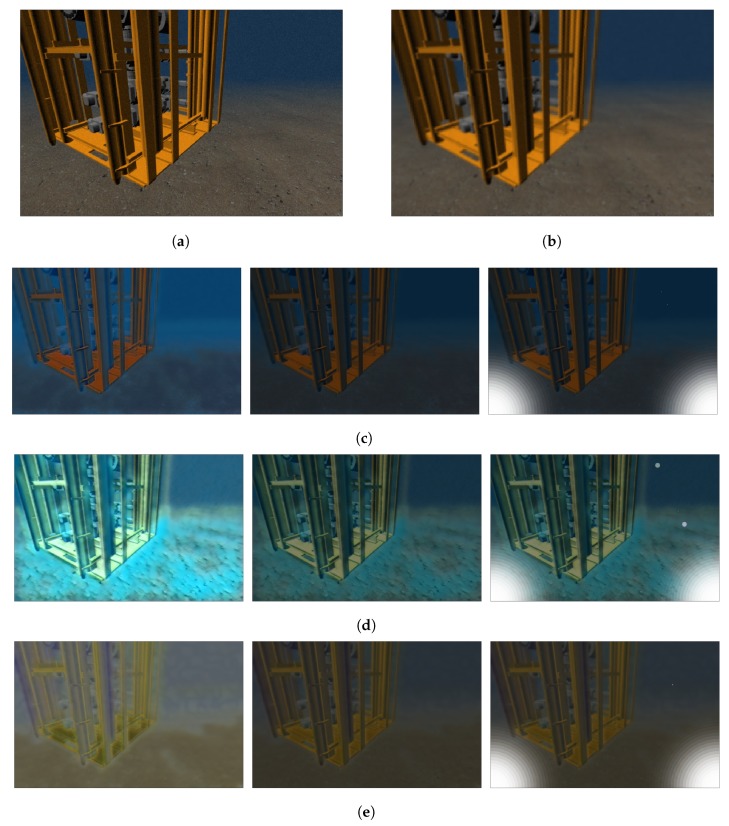
Processing of the simulator image for different sea models. (**a**) Original image from the UUV Simulator. (**b**) Forward-scattered image. (**c**) Atlantic ocean style, (**e**) Mediterranean sea style and (**d**) Muddy water style, with (from left to right) superposition value α=1, α=0.4 and with addition of two light sources.

Three water models (referred as UWmodel in Algorithm 1) have been currently trained. An Atlantic Ocean model (see Figure 6c), a Mediterranean sea model (Figure 6d) and a model with muddy water (Figure 6). The difference between them, and their similarities with the water types introduced in Figure 6 can be appreciated. Another interesting feature of applying style transfer to the synthetic images is the simulation of suspended particles and mud in the scene, which cannot be directly added to the simulator. Also, an α parameter has been implemented, as a superposition parameter that gives more weight to either the original or the styled image. This allows further customization of the resulting image without requiring any additional model training. The implementation of the α parameter is explained in Algorithm1, and the result of modifying it can be appreciated in Figure 6.

#### 3.2.3. Haze Addition

The presence of haze caused by artificial lights in underwater images presents a key issue in computer vision algorithms, as it affects the contrast on the image and occludes information on the scene. This is a particularly important condition in simulation for the development of visual navigation algorithms, which require tracking of features on the scene.

The algorithm incorporates an optional and configurable solution, which allows putting any desired number of light sources with any size or position. An RGBA image gauss_flare is generated as a Gaussian kernel according to the amount, color, position and size of the lights, and overlaid onto the style transferred image.

An example result is shown in Figure 5d, where two light sources in the bottom corners of the image are added. These light spots generate two bright saturated areas, which leads to a reduction of contrast in the background image.
**Algorithm 1** Underwater Image Simulation1:**procedure**uw_img_sim($I_{object}$, UWmodel, α, lights)2:    **Underwater Image Formation:**3:    **if** *there is* depth image $I_{Z}$ **then**4:        $I_{c}\leftarrow Attenuation\left(I_{object},I_{Z}\right)$5:        $F_{c}\leftarrow ForwardScatter\left(I_{c},I_{Z}\right)$6:    **else**7:        $F_{c}\leftarrow I_{object}$8:    **end if**9:    **Style Transfer:**10:    $I_{style}\leftarrow StyleTransfer\left(F_{c},UWmodel,\alpha \right)$11:    **Haze addition:**12:    **if** lights **then**13:        $I_{uw}\leftarrow Haze\left(I_{style},lights\right)$14:    **end if**15:    **return** underwater image $I_{uw}$16:**end procedure**17:**function**StyleTransfer(Img, model, α)18:    $I_{model}\leftarrow StyleModel\left(Img,model\right)$19:    $I_{\alpha }\leftarrow I_{model}\cdot \alpha +F_{c}\cdot \left(1-\alpha \right)$20:    **return** $I_{\alpha }$21:**end function**22:**function**Haze(Img, lights)23:    **for** light **in** lights **do**24:        gauss_flare←GaussianKernel(Img,light_pose,light_size)25:        $I_{haze}\leftarrow Img\cdot gauss\_flare$26:    **end for**27:    **return** $I_{haze}$28:**end function**

## 4. Proposed Algorithm for Image Enhancement

It is not convenient to directly employ the images obtained from either real or simulated environments for computer vision techniques, and thus is required to perform some prior processing on them. An enhancement approach has been selected, as it does not require prior knowledge or training of a model, and can be effectively applied in any of the given (real or synthetic) scenarios.

The proposed algorithm is hereafter referred as HSCM, according to its four processing steps:

1. **H**omomorphic filtering: it removes multiplicative noise, and improves the image by simultaneous intensity range compression (illumination) and contrast enhancement (reflection) [47].
2. **S**moothing: a median filter is used to smooth the image while preserving the edges.
3. **C**ontrast enhancement, with Contrast Limited Adaptive Histogram Equalization (CLAHE).
4. **M**ask application, for removing useless areas of the image.

**Figure 7:**
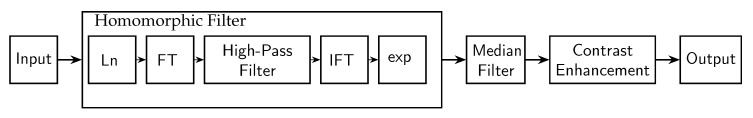
HSCM algorithm for underwater image enhancement.

The full algorithm is shown in Figure 7, and each of its mentioned steps are detailed hereafter.

### 4.1. Homomorphic Filtering

Homomorphic filtering is a generalized technique for image enhancement in bad illumination conditions. The image is modelled according to the product of the illumination and the reflectance:

(10) m(x,y)=i(x,y)•r(x,y)

where m(x,y) is the image provided by the camera, i(x,y) the illumination, and r(x,y) the reflectance function. It is assumed that the illumination changes slowly through the field of view, therefore it represents low frequencies in the Fourier Transform of the image. The reflectance, on the other hand, is the non-uniform illumination in the image, and thus is associated with high frequencies. By multiplying these components by any high-pass filter, the low frequencies can be suppressed along with the non-uniform illumination in the image.

According to this model, the image needs to be converted to the frequency domain to apply a high-pass filter. To simplify the calculations, logarithms are used, so the product can be expressed as a sum:

(11) ln(m(x,y))=ln(i(x,y))+ln(r(x,y))

Now, to express the log-image on the frequency domain, the Fourier Transform (FT) is applied:

(12) F(ln(m(x,y)))=F(ln(i(x,y)))+F(ln(r(x,y)))

which is also expressed as:

(13) M(u,v)=I(u,v)+R(u,v)

The high-pass filter selected, makes the illumination more even by increasing mid and high-frequency components (reflectance) and decreasing low-frequency components (illumination).

(14) N(u,v)=H(u,v)•M(u,v)

where H(u,v) is the high-pass filter and N(u,v), the filtered image in the frequency domain.

The high-pass filter, according to [43], is computed as:

(15) $$H\left(u,v\right)=\left(r_{H}-r_{L}\right)\left(1-\exp\left(-\frac{u^{2}+v^{2}}{2\delta^{2}}\right)\right)+r_{L}$$

where $r_{H}=2.5$ and $r_{L}=0.5$ are the maximum and minimum coefficients values and δ a factor which controls the cutoff frequency. They are selected experimentally.

**Figure 8:**
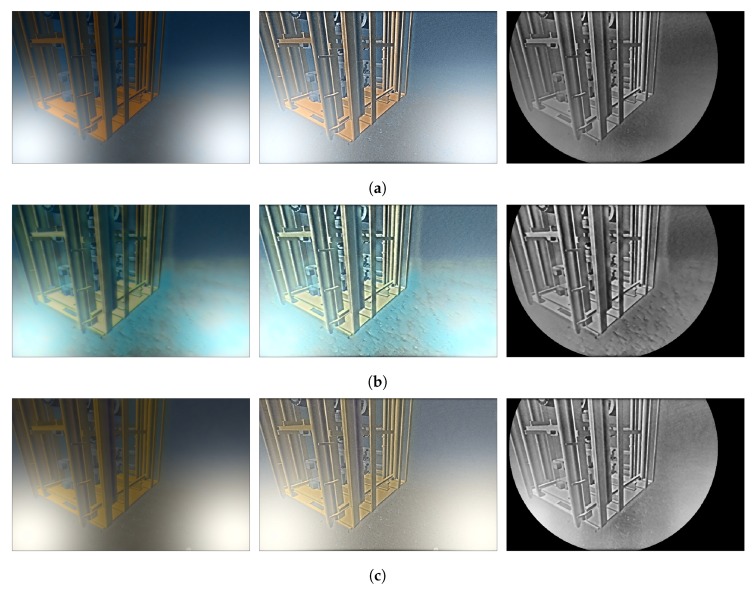
Image enhancement over the simulated underwater images for the (**a**) Atlantic Ocean, (**b**) Mediterranean sea, and for the (**c**) muddy water. From left to right: original image, homomorphic filtered image, and final result after applying the mask.

Finally, the filtered image is converted back to the spatial domain with the Inverse Fourier Transform (IFT), and the logarithm is reversed by computing the exponential. The result of applying the homomorphic filter can be seen in Figure 8.

### 4.2. Smoothing

The result of applying a homomorphic filter returns a noisy image, so a smoothing step is required to reduce it. A median filter is used as it efficiently removes impulsive noise such as the suspended particles while preserving the edges.

### 4.3. Contrast Enhancement

The low contrast of underwater images makes necessary to perform a contrast enhancement process. The artificial illumination generates a no uniform light distribution, with areas of very much brightness and areas of complete darkness. The algorithm selected then is the Contrast Limited Adaptive Histogram Equalization (CLAHE) [48]. This algorithm divides the image into regions of the desired size, and then computes the histogram of each region to later equalize it. This can amplify the noise, so contrast limiting is applied to every neighborhood point. A default value for the squared kernel size of 8×8 pixels has proven to provide good performance in the example images, which have a resolution of 869×565 pixels.

### 4.4. Mask

A circled mask that removes the useless areas of the image is applied. It removes the areas saturated by the light source, and the fixed parts of the UUV, which would induce error in the movement reconstruction of the image, as they appear fixed. A circled mask that removes the mentioned areas without generating false keypoints is applied to the image.

## 5. Results

In the present work, a simulation algorithm has been developed which emulates the underwater imaging conditions without requiring a large database or complex measurements of the environment to replicate. The framework also includes an image enhancement algorithm, which optimizes the extraction of features from the image for any given environment. The results for each of the components of the framework are detailed in this section.

### 5.1. Underwater Image Simulation

The simulation algorithm combines the modelling of the imaging conditions that are given in any underwater environment, with the use of Style Transfer techniques that provide the specific characteristics of the desired underwater environment.

An example of the algorithm working over an image from the Gazebo simulator is shown in Figure 6. The UUV Simulator, with its built-in light attenuation module, provides the image shown in Figure 6a, on which the forward scattering is applied afterwards, leading to the result from Figure 6b. These first two steps provide attenuation and scattering of light directly proportional to the depth of the object in the scene, and they are two common steps for any given underwater environment. The next step is the application of style transfer on the forward-scattered image, with a result dependent on the model used. Here, three models have been trained: An Atlantic ocean model (Figure 6c), a Mediterranean sea model (Figure 6d) and a muddy water model (Figure 6e). The differences for each model are mainly appreciated on the different coloring conditions that each environment provides, but another interesting feature is the presence of mud in the water. This is particularly convenient since Gazebo only admits well-defined shapes, and the presence of dust is one of the key problems on feature detection in underwater images. Moreover, a superposition value α has been implemented that gives more weight to either the forward-scattered image or to the style transferred image. This allows extra customization of the simulated image without requiring the training of a new model. Finally, another common effect on underwater images has been implemented, as it is the presence of hazes in the image due to the artificial lighting. An example case of two lights on each side of the image is shown for each underwater model in Figure 6c–e.

**Figure 9:**
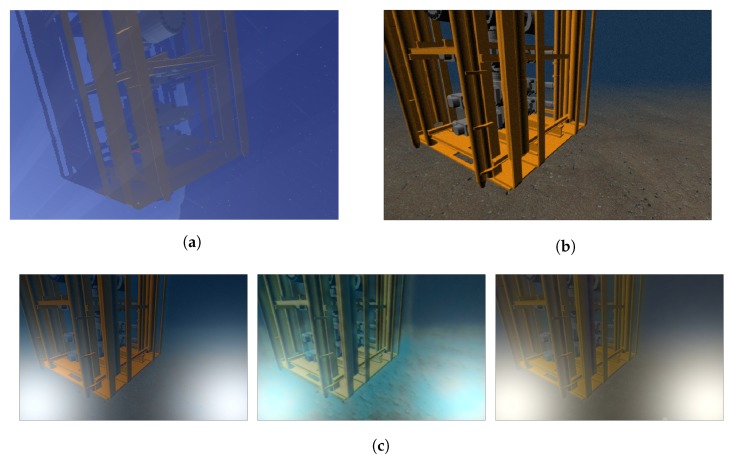
Simulation of the same underwater scene in the (**a**) UWSim, (**b**) the UUV Simulator, and (**c**) the proposed underwater simulator.

In Figure 9, the resulting images are compared with the images obtained by the two most popular underwater simulators: UWSim and UUVSim. UUVSim only performs a light attenuation with distance, while UWSim provides a more realistic scenario by adding water particles and sunlight reflections. UWSim also provides a few parameters (attenuation, color and density) to change the water appearance. However, the proposed approach can easily provide a more realistic simulation. It only requires the selection of the trained model that adds the color, attenuation or dust associated with the given environment. Moreover, it performs a forward scattering according to the object distance, and can add artificial lights to the scene.

### 5.2. Underwater Image Enhancement

**Figure 10:**
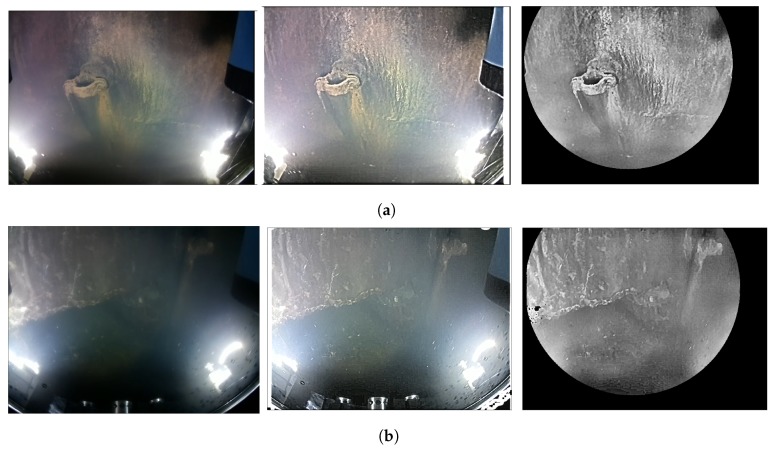
(**a**,**b**) Image enhancement over two real underwater frames. From left to right: original image, homomorphic filtered image, and final result after applying the mask.

The enhancement algorithm HSCM has been tested in the real and simulated environments (Figure 8 and Figure 10 respectively). For all cases, it can be appreciated how the homomorphic filter is able to provide a uniform illumination in the scene and emphasize the edges, despite the critical conditions of having two light sources in the field of view. After denoising the image, improving the contrast, and applying the mask, the features of the scene are emphasized while the areas that do not provide information (like those saturated by the artificial lights) are removed.

**Figure 11:**
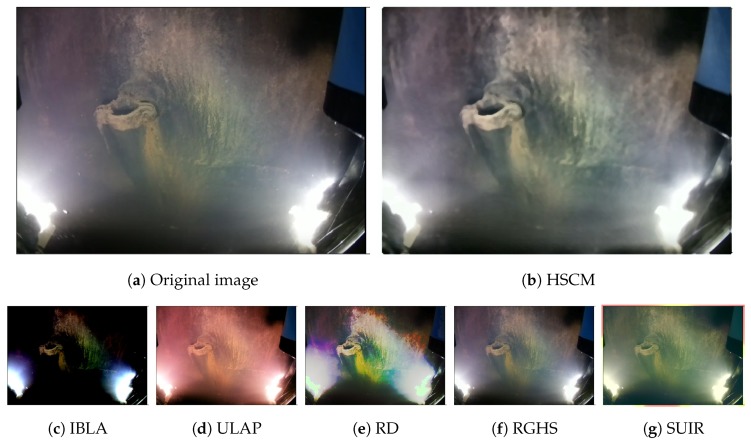
The different enhancement and restoration methods applied to a real underwater image.

The proposed solution HSCM has been compared to other state-of-the-art algorithms based on both restoration and enhancement: IBLA [40], ULAP [41], RD [46], RGHS [45] and SUIR [42]. The subjective analysis can be performed with the example images shown in Figure 11. Please note that the mask of the proposed algorithm has been removed in this Figure for a better comparison with the others. The restoration algorithms do not provide a correct color correction, as they have been designed for a specific image formation model. The results of the enhancement algorithms are closer to HSCM. However, our proposal provides a more uniform illumination of the scene.

The objective analysis is performed according to the following metrics:

- Entropy [49]: the abundance of information observed from the image. A higher value implies a more uniform contrast, and thus a better quality of the image.
- UICM (Underwater Image Colorfulness Measure) [50]: measures the quality according to the saturation and attenuation of each color. A greater value implies a better enhancement.
- UISM (Underwater Image Sharpness Measure) [50]: measures the preservation of the edges and details, with a better sharpness associated to a greater value.
- UIConM (Underwater Image Contrast Measure) [50]: provides a greater value for a better contrasted image.
- UIQM (Underwater Image Quality Measure) [50]: this value is obtained as a linear combination of the previous three, and similarly, a greater value implies a better enhancement.

These multiple metrics allow consideration of the information richness of the image. The results for each metric are shown in Table 1. It can be seen that the overall performance of our algorithm, provided by the UIQM parameter, is close to the maximum value. The same happens with entropy, which is an important parameter to consider for the development of visual navigation algorithms. Our algorithm outperforms the rest in terms of speed, being more than three times faster than the second fastest algorithm. It should be also noted that our algorithm provides the highest stability in the metric values, since some of the other algorithms provide higher standard deviations.

## 6. Conclusions and Future Works

This paper proposes a framework that contributes significantly to the development of underwater visual algorithms, as the following goals have been achieved:

- Development of an open-source tool for underwater imaging simulation.
- Integration of the proposed framework within a robotic simulator.
- Simulation of a realistic underwater environment from a single image model with the use of style transfer.
- Image enhancement for a greater information richness on the image.

The novelty of the simulation algorithm lies in the use of style transfer to model the imaging conditions of the particular underwater environment. Previous works perform complex measurements or require large datasets of the environment to be able to replicate its specific imaging conditions. The main contribution of the proposed algorithm is the ability to replicate any environment with the use of a single image from the desired environment. Three models have been trained for three different waters: the Atlantic Ocean, the Mediterranean Sea, and muddy water. This showcases the potential of the proposed algorithm, where each simulated environment is clearly differentiated from the others, providing a realistic output for each case.

The underwater image enhancement algorithm has proven to improve the contrast without increasing the noise that the image suffers in underwater conditions, which is more critical in muddy waters where the corners get more blurred. Moreover, the uneven lighting effects caused by artificial lights have been corrected with a homomorphic filter, followed by a mask that removes the areas of the image which do not contain information. This is especially convenient for the performance of visual localization algorithms, or for any process that requires the extraction of features in the image. Moreover, the similarity in the results obtained from the real images proves the ability of the algorithm to work in any environment provided.

Future works for this paper include the use of the proposed image simulator for two different tasks: (1) training a visual navigation algorithm, and hereafter check its performance in a real scenario. (2) training a DNN for image enhancement, to later compare the performance between the trained filter and the homomorphic filter. Moreover, the development of a metric for image enhancement that measures the improvement of light uniformity in the image would be an interesting contribution.

## Figures and Tables

**Table 1:** Image Quality Metrics applied to the images processed with different methods. The values are expressed as the mean with the standard deviation between parentheses.

Methods		Image Quality Metrics					
		Time [s]	Entropy	UICM	UISM	UIConM	UIQM
Image restoration	SUIR	7.080 (0.13)	10.14 (0.19)	4.42 (1.50)	2.45 (0.17)	**−0.12 (0.05)**	1.02 (0.09)
	IBLA	14.00(1.41)	4.62 (0.43)	5.59 (3.39)	2.24 (0.12)	−0.65 (0.44)	1.17 (0.16)
	ULAP	1.01 (0.01)	10.49 (0.23)	**5.79 (0.91)**	2.62 (0.17)	−0.61 (0.46)	**1.29 (0.18)**
Image enhancement	RD	3.54 (0.18)	10.77 (0.14)	5.29 (1.32)	**2.67 (0.34)**	−0.55 (0.40)	1.27 (0.25)
	RGHS	2.28 (0.11)	**10.49 (0.13)**	3.94 (0.93)	2.42 (0.14)	−0.63 (0.48)	1.17 (0.18)
	**HSCM**	**0.33 (0.01)**	10.42 (0.09)	3.09 (0.71)	2.56 (0.18)	−0.23 (0.39)	1.05 (0.17)

## Abbreviations

The following abbreviations are used in this manuscript:

UUV	Unmanned Underwater Vehicle
STAMS	Long-Term Stability Assessment and Monitoring of Flooded Shafts
RFCS	Research Fund for Coal and Steel
UWSim	UnderWater SIMulator
UUV Simulator	Unmanned Underwater Vehicle Simulator
PSF	Point Spread Function
DNN	Deep Neural Networks
VGG	Visual Geometry Group
ROS	Robot Operating System
CNN	Convolutional Neural Network
GAN	Generative Adversarial Network
IBLA	Image Blurriness and Light Absorption
ULAP	Underwater Light Attenuation Prior
SUIR	Single Underwater Image Restoration
RGHS	Relative Global Histogram Stretching
RD	Rayleigh Distribution
FT	Fourier Transform
IFT	Inverse Fourier Transform
CLAHE	Contrast Limited Adaptive Histogram Equalization
BRISQUE	lind/Referenceless Image Spatial Quality Evaluator
UICM	Underwater Image Colorfulness Measure
UISM	Underwater Image Sharpness Measure
UIConM	Underwater Image Contrast Measure
UIQM	Underwater Image Quality Measure

## References

[1] Weidner N., Rahman S., Li A.Q., Rekleitis I. Underwater cave mapping using stereo vision. Proceedings of the IEEE International Conference on Robotics and Automation.

[2] Hernández J.D., Istenic K., Gracias N., García R., Ridao P., Carreras M. (2016). Autonomous seabed inspection for environmental monitoring. Robot 2015: Second Iberian Robotics Conference.

[3] Johnson-Roberson M., Bryson M., Friedman A., Pizarro O., Troni G., Ozog P., Henderson J.C. (2017). High-resolution underwater robotic vision-based mapping and three-dimensional reconstruction for archaeology. J. Field Robot..

[4] Ozog P., Carlevaris-Bianco N., Kim A., Eustice R.M. (2016). Long-term Mapping Techniques for Ship Hull Inspection and Surveillance using an Autonomous Underwater Vehicle. J. Field Robot..

[5] Bonnin-Pascual F., Ortiz A. (2019). On the use of robots and vision technologies for the inspection of vessels: A survey on recent advances. Ocean. Eng..

[6] Ferrera M., Moras J., Trouvé-Peloux P., Creuze V. (2019). Real-Time Monocular Visual Odometry for Turbid and Dynamic Underwater Environments. Sensors.

[7] Jiang M., Song S., Li Y., Jin W., Liu J., Feng X. (2019). A Survey of Underwater Acoustic SLAM System. Proceeindgs of the International Conference on Intelligent Robotics and Applications, Shenyang, China, 8–11 August.

[8] Wang Y., Zhang J., Cao Y., Wang Z. A deep CNN method for underwater image enhancement. Proceedings of the IEEE International Conference on Image Processing (ICIP).

[9] Li J., Skinner K.A., Eustice R.M., Johnson-Roberson M. (2017). WaterGAN: Unsupervised generative network to enable real-time color correction of monocular underwater images. IEEE Robot. Autom. Lett..

[10] Oleari F., Kallasi F., Rizzini D.L., Aleotti J., Caselli S. An underwater stereo vision system: from design to deployment and dataset acquisition. Proceedings of the Oceans’15 MTS/IEEE.

[11] Sanz P.J., Ridao P., Oliver G., Melchiorri C., Casalino G., Silvestre C., Petillot Y., Turetta A. (2010). TRIDENT: A framework for autonomous underwater intervention missions with dexterous manipulation capabilities. IFAC Proc. Vol..

[12] Duarte A., Codevilla F., Gaya J.D.O., Botelho S.S. A dataset to evaluate underwater image restoration methods. Proceedings of the OCEANS.

[13] Ferrera M., Moras J., Trouvé-Peloux P., Creuze V., Dégez D. (2018). The Aqualoc Dataset: Towards Real-Time Underwater Localization from a Visual-Inertial-Pressure Acquisition System. arXiv.

[14] Akkaynak D., Treibitz T., Shlesinger T., Loya Y., Tamir R., Iluz D. What is the space of attenuation coefficients in underwater computer vision?. Proceedings of the IEEE Conference on Computer Vision and Pattern Recognition.

[15] Prats M., Perez J., Fernández J.J., Sanz P.J. An open source tool for simulation and supervision of underwater intervention missions. Proceedings of the IEEE/RSJ InternationalConference on Intelligent Robots and Systems.

[16] Manhães M.M.M., Scherer S.A., Voss M., Douat L.R., Rauschenbach T. UUV simulator: A gazebo-based package for underwater intervention and multi-robot simulation. Proceedings of the OCEANS 2016 MTS/IEEE.

[17] Matsebe O., Kumile C., Tlale N. (2008). A review of virtual simulators for autonomous underwater vehicles (auvs). IFAC Proc. Vol..

[18] Cook D., Vardy A., Lewis R. A survey of AUV and robot simulators for multi-vehicle operations. Proceedings of the IEEE/OES Autonomous Underwater Vehicles (AUV).

[19] Boeing A., Bräunl T. (2006). SubSim: An autonomous underwater vehicle simulation package. Proceedings of the 3rd International Symposium on Autonomous Minirobots for Research and Edutainment (AMiRE 2005).

[20] Koenig N., Howard A. Design and use paradigms for gazebo, an open-source multi-robot simulator. Proceedings of the IEEE/RSJ International Conference on Intelligent Robots and Systems (IEEE Cat. No. 04CH37566).

[21] Rohmer E., Singh S.P., Freese M. V-REP: A versatile and scalable robot simulation framework. Proceedings of the IEEE/RSJ International Conference on Intelligent Robots and Systems.

[22] Razzanelli M., Casini S., Innocenti M., Pollini L. (2019). Development of a Hybrid Simulator for Underwater Vehicles With Manipulators. IEEE J. Ocean. Eng..

[23] Jaffe J.S. (1990). Computer modeling and the design of optimal underwater imaging systems. IEEE J. Ocean. Eng..

[24] Cheng C.Y., Sung C.C., Chang H.H. Underwater image restoration by red-dark channel prior and point spread function deconvolution. Proceedings of the IEEE International Conference on Signal and Image Processing Applications (ICSIPA).

[25] Han P., Liu F., Yang K., Ma J., Li J., Shao X. (2017). Active underwater descattering and image recovery. Appl. Opt..

[26] Barros W., Nascimento E.R., Barbosa W.V., Campos M.F. (2018). Single-shot underwater image restoration: A visual quality-aware method based on light propagation model. J. Vis. Commun. Image Represent..

[27] Akkaynak D., Treibitz T. A revised underwater image formation model. Proceedings of the IEEE Conference on Computer Vision and Pattern Recognition.

[28] Sedlazeck A., Koch R. (2011). Simulating Deep Sea Underwater Images Using Physical Models for Light Attenuation, Scattering, and Refraction.

[29] Berman D., Levy D., Avidan S., Treibitz T. (2018). Underwater single image color restoration using haze-lines and a new quantitative dataset. arXiv.

[30] Ancuti C.O., Ancuti C., De Vleeschouwer C., Bekaert P. (2017). Color balance and fusion for underwater image enhancement. IEEE Trans. Image Process..

[31] Tenenbaum J.B., Freeman W.T. (2000). Separating style and content with bilinear models. Neural Comput..

[32] Simonyan K., Zisserman A. (2014). Very deep convolutional networks for large-scale image recognition. arXiv.

[33] Gatys L.A., Ecker A.S., Bethge M. (2015). A neural algorithm of artistic style. arXiv.

[34] Ulyanov D., Vedaldi A., Lempitsky V. (2016). Instance normalization: The missing ingredient for fast stylization. arXiv.

[35] Johnson J., Alahi A., Fei-Fei L. (2016). Perceptual losses for real-time style transfer and super-resolution. Proceedings of the European Conference on Computer Vision.

[36] Jing Y., Yang Y., Feng Z., Ye J., Yu Y., Song M. (2019). Neural style transfer: A review. IEEE Trans. Vis. Comput. Graph..

[37] Shin Y.S., Cho Y., Pandey G., Kim A. Estimation of ambient light and transmission map with common convolutional architecture. Proceedings of the OCEANS 2016 MTS/IEEE.

[38] Li C., Guo J., Guo C. (2018). Emerging from water: Underwater image color correction based on weakly supervised color transfer. IEEE Signal Process. Lett..

[39] Spier O., Treibitz T., Gilboa G. In situ target-less calibration of turbid media. Proceedings of the IEEE International Conference on Computational Photography.

[40] Peng Y.T., Cosman P.C. (2017). Underwater image restoration based on image blurriness and light absorption. IEEE Trans. Image Process..

[41] Song W., Wang Y., Huang D., Tjondronegoro D. (2018). A Rapid Scene Depth Estimation Model Based on Underwater Light Attenuation Prior for Underwater Image Restoration. Proceedings of the Pacific Rim Conference on Multimedia.

[42] Li C., Quo J., Pang Y., Chen S., Wang J. Single underwater image restoration by blue-green channels dehazing and red channel correction. Proceedings of the IEEE International Conference on Acoustics, Speech and Signal Processing.

[43] Bazeille S., Jaulin L., Quidu I., Malkasse J. Automatic Underwater Image Pre-Preprocessing. https://hal.archives-ouvertes.fr/hal-00504893/document.

[44] Iqbal K., Salam R.A., Osman A., Talib A.Z. (2007). Underwater Image Enhancement Using an Integrated Colour Model. IAENG Int. J. Comput. Sci..

[45] Huang D., Wang Y., Song W., Sequeira J., Mavromatis S. (2018). Shallow-water image enhancement using relative global histogram stretching based on adaptive parameter acquisition. Proceedings of the International Conference on Multimedia Modeling.

[46] Ghani A.S.A., Isa N.A.M. (2014). Underwater image quality enhancement through composition of dual-intensity images and Rayleigh-stretching. SpringerPlus.

[47] Weeks A.R. (1996). Fundamentals of Electronic Image Processing.

[48] Reza A.M. (2004). Realization of the contrast limited adaptive histogram equalization (CLAHE) for real-time image enhancement. J. VLSI Signal Process. Syst. Signal Image Video Technol..

[49] Wang Y., Song W., Fortino G., Qi L.Z., Zhang W., Liotta A. (2019). An Experimental-based Review of Image Enhancement and Image Restoration Methods for Underwater Imaging. IEEE Access.

[50] Panetta K., Gao C., Agaian S. (2015). Human-visual-system-inspired underwater image quality measures. IEEE J. Ocean. Eng..

